# Women’s heart health at mid-life: what is the role of psychosocial stress?

**DOI:** 10.1186/s40695-018-0041-2

**Published:** 2018-07-06

**Authors:** Andrea L. Stewart, Ummul-Kiram Kathawalla, Alexandra G. Wolfe, Susan A. Everson-Rose

**Affiliations:** 1 Department of Epidemiology, University of Pittsburgh, 4420 Bayard Street, Suite 600, Pittsburgh, PA 15260 USA; 2 Department of Psychology, University of Minnesota, 75 E River Parkway, Minneapolis, MN 55414 USA; 3 Department of Medicine, University of Minnesota, 717 Delaware St SE, Suite 166, Minneapolis, MN 55414 USA

**Keywords:** Cardiovascular disease, Discrimination, Psychosocial factors, Risk factors, Stress, Subclinical atherosclerosis, Women

## Abstract

**Background:**

Women in mid-life experience unique stressors, including transitions within their family roles, informal caregiving, job stress, and perceived discrimination. The impact of these stressors on cardiovascular health in women during mid-life is of growing interest in both the popular and scientific literature. The objective of this review is to summarize the recent literature on stress and cardiovascular health in mid-life women. We focus on stressors that are relevant to mid-life women, including social stress and discrimination, and long-term risk of CVD events and subclinical CVD measures.

**Methods:**

We systematically reviewed the literature published between January 2012 and April 2018 for studies examining stress in mid-life and either CVD endpoints or subclinical CVD outcomes. Eligible studies included at least one psychosocial stress exposure, a CVD or subclinical CVD outcome, and either included only female participants, reported sex-stratified analyses or tested for a sex*stress interaction.

**Results:**

We identified 37 studies published since 2012 that met our criteria and included women between the ages of 40 and 65, including 3 case-control studies, 15 cross-sectional studies, and 19 prospective cohort studies. Because clinical CVD events typically occur after age 65 in women, only 22 studies were available that evaluated stress and hard CVD events in samples with mid-life women. Results from these studies suggested an increased and significant risk of CVD due to stress. Of the 15 studies that included subclinical CVD outcomes, the majority showed that mid-life women experiencing greater levels of stress had more subclinical CVD, as indicated by carotid intima-media thickness, flow-mediated dilation and arterial stiffness; however, several studies reported null associations.

**Conclusions:**

General life stress, including perceived stress and life events, in mid-life was significantly related to later-life CVD risk and mid-life subclinical CVD in the majority of studies published in the past six years. Job stress was inconsistently related to CVD risk in women, and fewer studies examined characteristics of other social roles, such as marriage, motherhood or caregiving. Perceived discrimination also was associated with CVD events and subclinical CVD in some samples of mid-life women. Further investigation into specific stressors relevant to women in mid-life, including caregiving and marital stress, are needed to understand the full extent to which life stress impacts CVD risk in mid-life women.

## Background

Cardiovascular diseases (CVD), including coronary heart disease (CHD) and cerebrovascular disease, are the leading cause of death in women in the United States and many developing countries [1]. The United States population has experienced declines in heart disease mortality rates [2], but these declines have been observed mostly in older ages, with middle-aged and younger women seeing the least decline since 1990 [3]. Some projections even indicate that the trend for CHD mortality may reverse, with increases projected in middle-aged men and women by 2030 [4]. Additionally, racial disparities persist in CVD mortality and event rates among women, with black women experiencing higher mortality due to CVD, especially at younger ages [5]. Some common risk factors for CVD appear to impact men and women equally (e.g., elevated blood pressure and cholesterol), while others appear to be related to greater CVD risk in women than in men (such as diabetes and smoking) [6]. Studying CVD risk factors separately in men and women is important for understanding whether certain under-studied risk factors are more important in women.

Mid-life, the period of life between ages 40 and 65, may be a crucial time to study CVD and CVD risk in women, as this is a time when women are experiencing both physical and social changes associated with the transition from adulthood to older age and menopause. Recent studies of the menopausal transition show that the changes in lipids and vasomotor symptoms that occur during this period are related to subclinical CVD, a marker for later-life CVD risk [7, 8]. In addition to the physiologic changes that occur during mid-life, psychosocial factors in mid-life may play a role in women’s cardiovascular health, directly through biopsychosocial mechanisms, and by influencing their health behaviors.

Several reviews of the literature have examined psychosocial factors, including stress and life stressors, as predictors of heart disease specifically in women, finding evidence of potential effects of stress and stressful life events on increasing risk [9, 10]. Low and colleagues reviewed research from 1995 to 2009 regarding psychosocial risk and CHD in women and found that stress from relationships and family responsibilities may be more important than job stress alone for women’s cardiovascular health [10]. To our knowledge, no recent review of stress and CVD has focused specifically on women in mid-life. Furthermore, these prior reviews focused on studies of stress and heart disease diagnoses, events or mortality, which mostly occur in women in older age groups. In a review of prospective studies of chronic stressors and development of CHD published through 2011 [11], Steptoe and Kivimaki concluded that long-term stress relates to an approximately 50% excess risk of developing CHD. They also noted the feasibility of using non-invasive measures of subclinical CVD, such as carotid artery intima media thickness, in population studies to better understand the influence of stress on the atherosclerotic process, while acknowledging mixed results in that literature to-date. Their review did not address sex differences in effects of stress on the development of CVD over time.

Understanding the effect of psychosocial stress on subclinical CVD and clinical conditions known to increase the risk of CVD in mid-life can provide insight into pathways by which mid-life stressors impact later-life risk of heart disease and stroke. Identifying which stress exposures are most relevant to women’s health in mid-life also is important. Work-related stress has been extensively studied as a possible risk factor for CVD. High levels of job strain/work stress are related to poor cardiovascular health in women and men, but results of studies are mixed, and sex-specific effects of job stress on CVD health are unclear [12–15]. Notably, however, prior reviews of stress and CVD in women emphasized the need to examine the effects of stress from the other social roles that women occupy, such as relationships, parenting and caregiving for adult relatives, as well as the combinations of multiple roles [10]. There also has been recent interest in perceived discrimination as a unique stressor that may contribute to the excess CVD risk observed in ethnic minorities [16]. Women in mid-life can experience multiple forms of discrimination, including racism, sexism, and the beginning effects of ageism. In the Health and Retirement Study of adults over age 50, respondents aged 50–59 who completed the Everyday Discrimination Scale reported more experiences of unfair treatment than older age groups, and over 20% of respondents in this age group attributed their discrimination to age, or age and another attribution [17]. A 2014 review by Lewis and colleagues identified 34 studies of discrimination and CVD risk and risk factors between 2011 and 2013 but did not focus on women specifically. They concluded that there is a possible link between perceived discrimination and CVD risk, but that large, prospective, epidemiological studies with clinical endpoints are needed [16].

The main objectives of this review are: (1) to summarize the recent scientific literature since 2012 on the influence of stress in areas relevant to women in the transitional period of mid-life on cardiovascular health of women between the ages of 40 and 65; and (2) identify critical areas for future research that will promote greater understanding about heart health in women in their transitional middle years.

## Methods

We searched PubMed and PsychINFO databases for studies that were published between January 2012 and April 2018 that contained one of the terms “cardiovascular disease,” “heart disease,” “subclinical cardiovascular disease,” “heart failure,” “heart attack,” “myocardial infarction,” “stroke,” “atherosclerosis,” “intima media thickness” (and alternate spellings, i.e., “intimal medial thickness,” “intima media thickening,” “intimal medial thickening,”), “coronary artery calcification” (also “coronary artery calcium”), “aortic calcification,” “pulse wave velocity,” “endothelial function,” “plaque,” or “arrhythmia” and one of the terms “perceived stress,” “chronic stress”, “psychosocial stress,” “job stress,” “occupational stress,” “caregiver stress,” “marital stress,” “relationship stress,” “perceived discrimination,” “life events,” “psychosocial function,” or “psychosocial strain.” We used MeSH terms or Headings tools in PubMed and PsychINFO to restrict to peer-reviewed studies that were conducted in humans, written in English, and had female and middle-aged participants.

CVD events in mid-life women are rare, and few studies of exclusively mid-life women have enough statistical power to detect a significant difference in event risk. Furthermore, there is interest in understanding the physiologic pathways through which stress and psychosocial factors impact cardiovascular health in mid-life women prior to the development of clinical disease. Thus, we included studies of subclinical CVD outcomes, including carotid intima media thickness (cIMT), plaques, coronary artery calcification (CAC), endothelial function and arterial stiffness which are established indicators of CVD risk that can be studied in mid-life populations who experience few events.

**Fig. 1:**
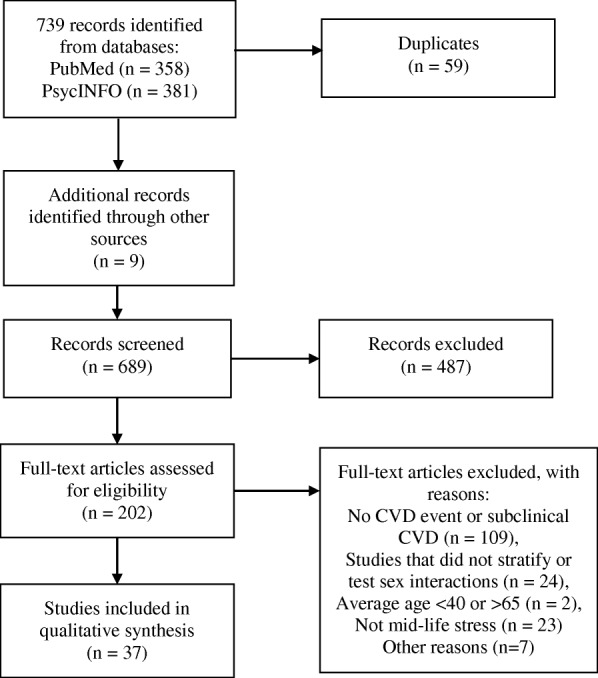
PRISMA flow diagram for studies included in review of stress and cardiovascular disease in mid-life women from January 2012 to April 2018

Our database searches returned 739 results, of which 59 were duplicates; 9 additional studies were identified by searching the references of these articles, for a total of 689 unique articles. The titles and abstracts were reviewed to determine if the studies met our review criteria: 1) the study sample included women in mid-life (i.e., between ages 40 and 65); 2) the study included a measure of at least one of our psychosocial stress factors of interest as an exposure; 3) the study outcomes included a measure of CVD or CVD risk. Title and abstract review eliminated 487 articles; we then reviewed the full text of the remaining 202 articles to determine if: 1) the psychosocial stress exposure or stressors occurred in mid-life, 2) the main outcome of the study was a CVD event or subclinical CVD, 3) the study sample included participants within the 40 to 65-year-old age range, and 4) one of the following was true: a) the population was exclusively female; b) analyses were stratified by sex; or c) an interaction of the stress exposure with sex was tested (using a cross product term in models). If a significant interaction between stress and sex was found in a study, we reported the results for the subsequent stratified analyses for women. The results reported were the main results from fully-adjusted models in each study. Because few studies were done in exclusively mid-life populations, we included studies whose population included women in mid-life (40–65) and for which the reported mean or median age was in that range, or that conducted age-stratified analyses with a mid-life age range as one of the stratum. This resulted in a total of 37 studies for inclusion in the review (see Fig. 1 for Preferred Reporting Items for Systematic Reviews and Meta-Analyses (PRISMA) diagram) [18]). A summary of the articles included in our review can be found in Tables 1 and 2.

**Table 1:** Results of Studies of Stress and Cardiovascular Disease Events in Mid-life Women

Author, Year, Study Name/Brief Description	N (% women)	Age Range	Mean Age (SD or SE)	Stratified by Sex or tested Stress X Sex Interaction^a^	Study design	Outcome	Stress measure	Results	QAT Rating
Bohley et al.*,* 2016, Population based study of East German adults [40]	3901 (49%)	20–83	Women: 48.8 (12.1)	Stratified	Cross-sectional	CHD (MI, CABG, PCI), and CVD (CHD or stroke) (Self-report)	Stressors related to German Reunification (financial, occupational, personal)	Reunification stress related to greater prevalence of CHD in women only.	Fair
Buyck et al., Whitehall II, 2013 [48]	7925 (31%)	39–63	49.5 (6.1)	Stratified	Prospective	CHD (fatal CHD, MI, angina) (Hospital record/self-report/verified with ECG)	Caregiving status, burden (> 5 vs < 5 h/week), duration	CHD risk did not differ between caregivers and non-caregivers. Caregiving burden and duration also not associated with greater CHD risk.	Fair
Dunlay et al.*,* 2017, Jackson Heart Study [50]	5085 (64%)	21–94	55.3 (12.8)	Interaction (n.s.)	Prospective	Incident CHD, stroke or heart failure (Adjudicated)	Everyday Discrimination, Lifetime Discrimination	No association between discrimination and CVD outcomes.	Good
Egido et al.*,* 2012, A case-control study of stress and stroke [41]	450 (50%)	18–65	53.8 (9.3)	Interaction (n.s)	Case-control	Stroke (Hospital record)	Holmes & Rahe Questionnaire of Life Events	Stroke patients had higher odds of stressful life events than neighborhood controls.	Good
Everson-Rose et al.*,* 2014, MESA [34]	6749 (53%)	45–84	62.1 (10.2)	Interaction (n.s.)	Prospective	Incident stroke, TIA (Adjudicated)	Chronic Burden Scale	Higher levels of chronic stress burden associated with increased risk of stroke or TIA.	Good
Everson-Rose et al.*,* 2015, MESA [51]	6508 (53%)	45–84	62 (10.2)	Interaction (n.s.)	Prospective	Incident CVD events (adjudicated)	Everyday Discrimination, Lifetime discrimination	Lifetime discrimination in ≥2 domains related to greater risk of CVD events. Everyday discrimination not related to CVD events in women.	Good
Gallo et al.*,* 2014, HCHS/SOL SCAS [39]	5313 (62%)	18–74	Not reported, 62% of sample age 45–74	Interaction (n.s.)	Cross-sectional	Prevalent CVD (Self-report)	Chronic Burden Scale, Perceived Stress Scale, Traumatic Stress Screener	Chronic stress, but not PSS or traumatic stress, significantly related to prevalent CHD.	Good
Graff et al., 2017, Danish National Health Survey [38]	114,337 (54%)	25+	Not reported, majority 45–65	Interaction (n.s.)	Prospective	Atrial Fibrillation (Patient register)	Perceived Stress Scale	No significant association between PSS and 4-year AF incidence after adjustment for comorbidities, SES and lifestyle factors.	Good
Guiraud et al., 2013, A case-crossover study of ischemic stroke and stress [42]	247 (42%)	18+	61.3 (15.9)	Interaction (n.s.)	Case-crossover	Stroke (Hospital record)	Interview for Recent Life Events	Stroke patients experienced ≥1 life event more often during first month and week preceding stroke than during control periods.	Fair
Jood et al., 2017, A case-control study of stress and stroke [45]	594 (32%)	30–65	54.4 (7.9)	Stratified	Case-control	Stroke (Hospital record)	Past 12-month job strain, Effort-reward imbalance (ERI), Work conflicts	Job strain, conflict at work, and ERI all higher in stroke cases than community controls.	Fair
Kershaw et al.*,* 2014, Women’s Health Initiative Study [32]	82,000 (100%)	52–72	Women: 62.1 (0.05)	N/A	Prospective	CHD and stroke (Adjudicated)	Life events, Social strain	Life events and social strain related to increased CVD, but not after adjusting for behavioral and biological risk factors.	Good
Kershaw et al., 2015, MESA [33]	6678 (50%)	45–84	62.4 (0.2)	Interaction (n.s.)	Prospective	Incident CHD (Adjudicated)	Chronic Burden Scale (Individual stress), Neighborhood-level stress	High tertile individual chronic stress and medium tertile neighborhood chronic stress associated with greater incident CHD.	Good
Kriegbaum et al., 2013, Danish Population Study [47]	42 million (50%)	30–65	Not reported, age range 30–65	Stratified	Prospective	MI (Registry)	Broken partnership	Broken partnerships were associated with increased risk of incident MI in the year of the break-up and in subsequent years. The association varied by age, with MI risk of same-year break-up significant among older women (50–65) but not younger women (30–49).	Good
Lazzarino et al., 2013, Health Survey for England [35]	66,518 (54%)	35+	55.1 (13.8)	Stratified	Prospective	CVD mortality (Vital registration)	Psychological distress	Greater psychological distress associated with risk of CVD death.	Good
Morton et al., 2014, MIDUS [49]	3032 (52%)	25–74	Not reported	Stratified	Prospective	MI (Self-report)	Family strain (demands, criticism, let down, get on nerves)	Family strain in mid-life not related to MI.	Fair
Ogilvie et al., 2016, MESA [36]	6809 (53%)	45–84	62.2 (10.2)	Interaction (n.s.)	Prospective	Heart failure (Adjudicated)	Chronic Burden Scale	High baseline chronic stress not related to increased risk of heart failure	Good
O’Neal et al., 2015, REGARDS [37]	25,530 (54%)	Not reported	65 (9.4)	Stratified	Cross-sectional	Atrial Fibrillation (ECG)	Perceived Stress Scale	PSS significantly related to AF, even after adjustment for covariates.	Fair
Padyab et al., 2014, Swedish Intervention Program [43]	74,988 (51%)	40–60	49 (8)	Stratified	Prospective	CVD mortality (Registry)	Karasek job strain model (demand, control)	Job demand, control, and job strain not associated with CVD risk.	Good
Redmond et al., 2013, REGARDS [31]	24,439 (55%)	Not reported	64.1 (9.3)	Interaction (n.s.)	Prospective	Incident CHD, Incident fatal CHD (Adjudicated)	Perceived Stress Scale	Participants with a stress score in the highest tertile had a greater risk of incident CHD, in low income group only (<$35,000).	Good
Slopen et al., 2012, Women’s Health Study [46]	22,086 (100%)	Not reported	Women: 57.2 (5.2)	N/A	Prospective	Incident CVD (MI, stroke, CABG, PCI or CVD death) (Adjudicated)	Job strain, Job insecurity	Job strain, but not insecurity, related to incident CVD. Women with “active” jobs (high demand, high control) also had an increased CVD risk.	Good
Svensson et al., 2017, Malmö Diet and Cancer Study [44]	18,559 (62%)	45–73	58.3 (8.0)	Interaction (n.s.)	Prospective	CAD (MI, CABG, PCI); fatal MI; non-fatal MI; CVD death (Government Registries)	Job strain, non-work stress	Combined work and non-work stress index unrelated to CAD, MI or CVD death.	Good
Udo et al., 2016, NESARC [52]	21,357 (53%)	18+	46.2 (16.4)	Stratified	Cross-sectional	self-reported MI, stroke, arteriosclerosis	Perceived weight discrimination, Stressful life events	Perceived weight discrimination associated with arteriosclerosis and MI in women only.	Poor

^a^In this column “n.s.” indicates that an interaction was tested in an analysis and found to be non-significant and results reported are for pooled estimates between men and women. An “s” indicates a significant interaction was found, and results reported are specific to women. For studies that reported sex-stratified analyses, only results for women are reported

**Table 2:** Results of Studies of Stress and Subclinical Cardiovascular Disease in Mid-life Women

Author, Year, Study Name/Brief Description	N (% women)	Age Range	Mean Age	Stratified by Sex or tested Stress X Sex Interaction ^a^	Study design	Outcomes	Stress measure	Results	QAT Rating
Bomhof-Roordink et al., 2015, Netherlands Study of Depression and Anxiety [58]	650 (65%)	20–66	46.5 (12.1)	Interaction (n.s.)	Cross-sectional	Carotid bifurcation IMT, Plaque presence, Arterial stiffness (Ultrasound/ Radial applanation tonometry)	Job strain, Negative life events	Recent life stress significantly associated with arterial stiffness but not IMT or plaque, even after adjusting for lifestyle factors.	Good
Camelo et al., 2015, ELSA-Brasil [64]	8806 (54%)	35–74	Not reported, 47.4% of sample between ages 45–54	Stratified	Cross-sectional	Carotid IMT (Ultrasound)	Job strain	High strain jobs associated with higher IMT, but not independent of life course SES. Passive work (low control, low demand) also associated with IMT in women.	Good
Charles et al., 2014, MESA [62]	1499 (45%)	45–84	56.1 (0.21)	Interaction (n.s.)	Cross-sectional	Flow-mediated dilation (Ultrasound)	Job strain	Job strain, demands, and control not associated with FMD. Occupational category related to FMD in women only.	Good
Flores-Torres et al., 2017, Mexican Teacher’s Cohort [56]	634 (100%)	40+	Women: 48.9 (4.3)	N/A	Cross-sectional	Carotid IMT, Plaques (Ultrasound)	Life Stressors Checklist (Physical or Sexual violence)	Exposure to physical violence associated with increased IMT and odds of subclinical carotid atherosclerosis. Longer duration of sexual violence associated with IMT. Violence in adulthood, but not childhood, associated with subclinical CVD.	Fair
Fujishiro et al., 2015, MESA [63]	3109 (52%)	45–84	Women:59.8 (9.4)	Stratified	Prospective	Carotid plaque, IMT progression (Ultrasound)	Job demands, Control, Interpersonal stress	Job strain, interpersonal stress not related to subclinical CVD progression in women.	Good
Gebreab et al., 2012, Jackson Heart Study [53]	3980 (63%)	21–94	Women:55.1 (12.5)	Stratified	Cross-sectional	Carotid plaque (Ultrasound)	Global Perceived Stress Scale, Holmes & Rahe Life Changes, Jones & Brantley Weekly Stress Inventory	Stress unrelated to presence of plaque in carotid arteries.	Fair
Janssen et al., 2012, SWAN [65]	336 (100%)	48–58	Women: 50.8 (2.7)	N/A	Prospective	CAC progression (Electron Beam Computed Tomography)	Multiple Role Questionnaire	Stress not related to CAC. Rewarding roles associated with CAC progression in Black women.	Good
Joseph et al. 2014, AHAB-2 [66]	281 (48%)	30–55	42.0 (7.3)	Interaction (n.s.)	Cross-sectional	Carotid IMT (Ultrasound)	Marital interaction quality	More negatively-rated interactions with spouses associated with greater IMT.	Good
Kamarck et al., 2012, Pittsburgh Healthy Heart Study [54]	270 (54%)	50–70	Not available	Interaction (n.s.)	Prospective	Plaque, IMT progression (Ultrasound)	Daily psychological demands	Increased demands in non-hypertensive patients associated with increased plaque, IMT.	Good
Kershaw et al., 2017, MESA [60]	2963 (53%)	45–84	61.8 (13.6)	Interaction (n.s.)	Cross-sectional	Flow-mediated dilation (Ultrasound)	Chronic Burden Scale	Chronic stress significantly related to %FMD, but not after adjusting for risk factors.	Good
Ortega-Montiel et al., 2015, GEA Study [57]	1243 (67%)	30–75	54.2 (9.0)	Stratified	Cross-sectional	Carotid IMT (Ultrasound)	Chronic self-perceived stress	Higher stress associated with greater IMT in women only.	Fair
Peterson et al., 2016, SWAN [67]	1056 (100%)	Not reported	Women: 59.48 (2.7)	N/A	Prospective	Carotid IMT, AD (Ultrasound)	Everyday Discrimination Scale	Cumulative unfair treatment associated with IMT in Caucasian but not minority women.	Good
Shah et al., 2016, MASALA] [59]	894 (47%)	40–84	Women: 54.4 (8.7)	Stratified	Cross-sectional	Carotid IMT (Ultrasound)	Chronic Burden scale, Everyday Discrimination	Current life stress and life stress over the past six months significantly related to CCA IMT in women only. Everyday discrimination unrelated to IMT in South Asian women.	Good
Thurston et al., 2014, SWAN [55]	1402 (100%)	Not reported	Women: 59.6 (2.7)	N/A	Cross-sectional	Carotid IMT, Plaque (Ultrasound)	Childhood physical/Sexual abuse; Adulthood physical/Sexual abuse	Adulthood abuse was related to greater odds of plaque; no associations with IMT.	Good
Thurston et al., 2018, peri- and post-menopausal women [61]	272 (100%)	40–60	Women: 54 (3.9)	N/A	Cross-sectional	Flow-mediated dilation (Ultrasound)	Brief Trauma Questionnaire, Childhood Trauma Questionnaire	Women with ≥3 trauma exposures in adulthood had worse (lower) FMD; association unchanged after controlling for childhood trauma.	Good

Acronyms: *AF* atrial fibrillation, *AHAB-2* Adult Health and Behavior Project- Phase 2, *CABG* Coronary artery bypass graft, *CAC* coronary artery calcification, *CHD* coronary heart disease, *CVD* cardiovascular disease, *ECG* Electrocardiogram, *ELSA-Brasil* Brazilian Longitudinal Study of Adult Health, *ERI* effort-reward imbalance, *FMD* flow-mediated dilation, *GEA* Genetics of Atherosclerosis Disease [Genetica de la Enfermedad Aterosclerosa], *HCHS/SOL SCAS* Hispanic Community Health Study/Study of Latinos Sociocultural Ancillary Study, *IMT* intimal-media thickness, *MASALA* Mediators of Atherosclerosis in South Asians Living in America, *MESA* Multi-Ethnic Study of Adults, *MI* myocardial infarction, *n.s.* not significant, *PCI* percutaneous coronary intervention, *QAT* Quality Assessment Tool, *REGARDS* Reasons for Geographic and Racial Differences in Stroke, *SES* Socio-economic status, *SWAN* Study of Women’s Health Across the Nation, *TIA* transient ischemic attack

^a^In this column “n.s.” indicates that an interaction was tested in an analysis and found to be non-significant and results reported are for pooled estimates between men and women. An “s” indicates a significant interaction was found, and results reported are specific to women. For studies that reported sex-stratified analyses, only results for women are reported

The 4 co-authors of this review evaluated the 37 studies for quality using the Quality Assessment Tool (QAT) for Observational Cohort and Cross-Sectional Studies or the QAT for case-control studies, both developed by the National Heart, Lung and Blood Institute [19, 20]. The QAT utilizes a rating scale of ‘good’, ‘fair’, or ‘poor’ depending on 14 criteria to examine the key concepts of internal validity for each study. An overall rating reflects the potential of bias underlying the methods and presentation of data. Two authors reviewed each study independently. After the initial review, there was full agreement on QAT ratings for 2/3 of the articles; disagreements on the quality of the remaining articles were resolved through discussion of the articles among reviewers until consensus on quality was achieved.

## Measures of stress in mid-life

The studies identified using our search strategy and criteria included diverse measures of psychosocial stress in mid-life, with a focus on stressors that were relevant to women during this period. These measures included, but were not limited to, validated questionnaires and scales such as the Chronic Burden Scale [21], Perceived Stress Scale [22], Holmes & Rahe Life Events Scale [23, 24], Everyday and Lifetime Discrimination Scales [25, 26]. We targeted our search for studies of the effects of perceived stress, stressful life events, stress related to work, marriage and relationships, caregiving and family responsibilities, and perceived discrimination on cardiovascular health and disease risk in mid-life women. We recognize that early life stressors are important in examining cardiovascular health due to the chronic nature of CVD; however, the goal was to focus on stressful life events in midlife to specifically examine the unique physical and social changes occurring for women during their transitional middle years and associations of these stress exposures with CVD risk. We acknowledge the importance of considering stressors earlier in life, as they may moderate the mid-life stress-CVD relationship, however, studies primarily examining early life stress were outside the scope of our review.

### General stress measures

Significant heterogeneity in measures of stress was observed in our final sample of studies. Studies that used at least one general stress measure included Cohen’s Perceived Stress Scale [22], inventories of stressful life events [23, 24] and the Chronic Burden Scale [21] which assesses the presence and severity of ongoing stress in 5 domains: one’s own health problems, health problems of close others, job or ability to work, relationships, and finances, and various other single- and multiple-item surveys asking about recent and chronic perceived psychological stress. Similarly, there was substantial variability in the studies of job-related stress, with some using the job strain model proposed by Karasek [27], some using the effort-reward imbalance model [28], and others using other scales or questions to assess job-related stress or stressors. Stress due to other social roles (family, marriage and relationships, and caregiving) was also conceptualized using a diverse set of scales and also by using objective stressors (i.e. relationship breakup from administrative records).

Finally, perceived discrimination was predominantly measured using the Everyday Discrimination Scale and the Lifetime Discrimination Scale in the studies found. These scales do not attribute discriminatory experiences to a specific characteristic but ask participants to report the main factors perceived as being the reason for the discrimination they reported [25, 26]. Thus, studies that used these scales either used the overall summary without attribution or restricted to sub-populations who indicated the discrimination was due to a specific trait (for example, in a study of weight discrimination limited to overweight or obese participants). Studies also measured perceived discrimination by the Schedule of Racist Events (SRE) [29] and Jackson Heart Discrimination Instrument [30], which measured everyday and lifetime discrimination, as well as the burden of lifetime discrimination and the effect of skin color. These were used exclusively in African-American populations.

Because various measures of stress were used, for ease of presentation, we have organized our review by outcomes. The first section includes studies of stress and CVD events, such as myocardial infarction, stroke, revascularization, mortality. The second section looks at the effects of stress on subclinical CVD. Within each outcome section, we further organize the presentation of findings by three categories based on the stress exposures: 1) general stress measures, 2) social role-related stressors, and 3) discrimination.

After reviewing all 37 articles using the QAT, 27 articles were rated ‘good’, 9 were rated ‘fair’, and one was rated ‘poor’ (see Tables 1 and 2). Overall, about 2/3 of all articles showed positive associations between stress/stressor and CVD or subclinical CVD.

## Cardiovascular disease outcomes: Clinical events

### CVD events and general stress measures

Twelve studies with CVD events or diagnoses as outcomes were included in our final sample of papers utilizing general measures of psychosocial stress. Seven studies were longitudinal follow-up studies, three were cross-sectional studies, one was a case-control study and one was a case-crossover study. The majority of these studies (83%) showed a positive relation between general stress exposures and increased CVD risk; of these seven were ‘good’ quality and three were ‘fair’ according to the QAT ratings. Among the studies with null findings, both were rated ‘good’.

Among the longitudinal studies, perceived stress [31], stressful life events [32], social strain [32], chronic neighborhood and individual stressors [33], chronic stress burden [34], and psychological distress [35] were associated with increased risk of CVD onset, events or mortality in cohorts ranging in size from 6105 to 82,107. The only null finding among these studies was by Ogilvie and colleagues, who found no relationship between chronic stress burden and heart failure onset in the Multi-Ethnic Study of Atherosclerosis (MESA) [36]. All but one of the longitudinal studies was conducted in cohorts that included men and women. The study focused on women [32] used data from the Women’s Health Initiative, a study of over 82,000 women ages 50 to 79, and demonstrated that social strain and stressful life events were associated with a significantly greater risk of CVD in minimally-adjusted models. These relationships became non-significant after adjusting for traditional CVD risk factors (including alcohol use, smoking, physical activity, diet, waist circumference, diabetes, hypertension and high cholesterol) [32]. None of the studies that included both women and men identified any sex differences in the relation of the stress exposure and CVD events [31, 33–36]. All but one study assessed stress at a single point in time only; Everson-Rose and colleagues found that using time-varying measures of chronic stress at two time points during follow-up resulted in a slightly larger increased hazard of incident stroke and transient ischemic attack than if baseline stress was used alone [34]. We identified two studies that examined the relationship between perceived stress and the presence or development of atrial fibrillation (AF). Perceived stress (according to the Perceived Stress Scale) was associated with electrocardiogram-confirmed AF in a cross-sectional study in the US [37], but in a Danish longitudinal survey, baseline PSS was not related to long-term risk of AF [38].

In two cross-sectional studies of diverse populations, past stressful life events and chronic stress burden were associated with self-reported diagnoses of heart disease and stroke prevalence. Gallo and colleagues found that chronic stress burden in a major life domain for six months, was associated with prevalent CHD and stroke in a Hispanic population in the United States [39]. In a cohort study of Germans, a greater perceived change in stressors as a result of German reunification (worse financial, occupational and personal situation) was related to greater odds of having a self-reported CVD diagnosis in women [40]. In addition to the two cross-sectional studies, the case-control and case-crossover studies demonstrated greater odds of stressful life events prior to stroke in middle aged populations that included women [41, 42].

Of the 12 papers reviewed that studied CVD events and general stress measures, all 5 of the cross-sectional, case-control, and case-crossover studies showed associations between stress and heart disease, stroke, or CVD, whereas five of seven (71%) longitudinal studies reported positive associations with increased CVD risk or presence/development of AF.

### CVD events and social role-related stress and stressors

Stress from jobs, partnership, caregiving, family strain or social relations were examined as predictors of incident CVD events in seven studies, with mixed findings depending on exposure measurement and population. Three of these studies showed a positive relation between social role-related stress/stressors and increased CVD risk, of these two were ‘good’ and one was ‘fair’ according to the QAT ratings. Among the four studies with null findings, two were rated ‘good’ and two ‘fair’. Job strain was unrelated to cardiovascular mortality among employed middle-aged women in two Swedish cohorts [43, 44]. In a Swedish case-control study, stroke cases had higher job strain, effort-reward imbalance and interpersonal conflicts at work than controls [45]. In the American Women’s Health Study, high strain and “active” jobs (high demand and high control), but not job insecurity, were associated with increased 10-year risk of CVD in female health professionals with an average age of 57 at baseline [46].

With regards to family and relationship stress, Kriegbaum and colleagues used population records in Denmark and showed that there was an increased risk of myocardial infarction in the years after a breakup of a partnership (defined as marriage or cohabitation) in middle aged adults, with the risk being highest for women under 40 in the year after the breakup, but for women over 50 the risk was highest in the same year as the breakup [47]. One study found that caregiving status and burden was unrelated to CHD development among middle-aged British civil servants [48]. In MIDUS, a study of mid-life in US adults, family strain, defined by perceived demands, criticism, disappointment, or bother from family members, was unrelated to MI incidence [49]. Overall, both partnership breakups and some type of job strain/stress showed positive associations with CVD [46, 47], however, caregiving and family strain were unrelated.

### CVD events and discrimination

The three studies of discrimination and CVD events yielded mixed findings; of these, two were ‘good’ and one was ‘poor’ according to the QAT rating scale. Two studies looked at the effects of both everyday discrimination (unfair treatment in day-to-day life), and lifetime discrimination (unfair treatment in a major domain of life such as a job, school or housing) on risk of CVD events and mortality. Everyday discrimination did not predict incident CHD, stroke or heart failure hospitalizations after adjusting for demographic, clinical, behavioral and socioeconomic variables in 11 years of follow-up in the Jackson Heart Study [50], or in sex-stratified analyses in the Multi-Ethnic Study of Atherosclerosis (MESA) [51]. Lifetime discrimination in the Jackson Heart Study did not predict incident CHD, stroke or heart failure [50], but was related to incident CVD in MESA, and controlling for chronic stress and depressive symptoms reduced, but did not eliminate this association [51]. Similarly, in a cross-sectional study, Udo et al. reported that experiencing lifetime discrimination due to weight was associated with a higher prevalence of self-reported MI, although controlling for stressful life events reduced the magnitude and significance of these relationships [52]. Although CVD events were not related to everyday discrimination [50, 51], they had a higher association with lifetime discrimination [51, 52].

### Summary: CVD events and stress

A wide range of studies have been published in the past 6 years examining the effects of perceived stress and stressful life events on CVD events in populations that include mid-life women. Over 3/4 of studies reviewed showed a positive relation between general stress exposures and increased CVD risk.

Stress, distress and stressful life events were related to CVD events in cross-sectional and prospective studies of women in mid-life populations or cohorts that included mid-life women. Two of four studies examining job stress, and one study examining breakup of partnership, an indicator of relationship stress, were predictive of increased risk of myocardial infarction in mid-life women [47]. Only one study examined job stress concurrent with stress from other social roles, using a single question to assess “stress or mental pressure because of problems or demands not related to your work” and found no significant effect of work or non-work stress on CVD [44]. Lifetime experiences of discrimination also predicted CVD in a national multi-ethnic cohort study of racial or ethnic minorities, but not in a study limited to African Americans in Jackson, Mississippi. Null findings in large longitudinal studies of heart failure and atrial fibrillation indicate that these are not likely pathways by which stress impacts health, although more studies are needed in other populations. These studies reflect a diverse set of stress exposures, which makes standardization of effect sizes difficult. Few studies compared the magnitude of the association between stress and CVD to known risk factors such as smoking and physical activity, but the ones that did found the effect size to be comparable.

## Subclinical cardiovascular disease

### Subclinical CVD and general stress measures

Subclinical CVD outcomes can be used in studies of mid-life populations to assess risk of CVD events and elucidate potential mechanisms by which stress in mid-life leads to clinically-relevant symptoms and events in later life. We identified nine studies of general stress exposures that included various indicators of subclinical CVD as outcomes, including carotid plaque [53–56], cIMT [54–59], endothelial function [60, 61], and central arterial stiffness [58]. Eight studies were cross-sectional and one study examined the effects of psychosocial demands on progression of subclinical CVD over a period of 6 years. A majority (78%) of these studies showed a positive relation between general stress exposures and increased subclinical CVD risk; among the studies reporting positive associations, five were ‘good’ and two were ‘fair’ according to the QAT ratings. Of the two studies with null findings, one was rated ‘good’ and one was rated ‘fair’.

In the studies reviewed, the most frequently studied subclinical CVD measure was cIMT, which was used in six studies, followed by carotid plaque presence, used in five studies. Most, though not all, of these studies reported positive findings. Negative life events and daily hassles were related to arterial stiffness, assessed by the augmentation index, but were unrelated to cIMT or plaque in the Netherlands Study of Depression and Anxiety [58]. In the Jackson Heart Study, a cross-sectional analysis of weekly stressors, past-year global perceived stress and negative life events found no significant associations with carotid plaque presence in women [53]. However, in a Mexican mestizo population, women who reported experiencing chronic stress for more than five years had thicker cIMT than non-chronically stressed women [57]. Chronic stress also was related to cIMT in women in the Mediators of Atherosclerosis in South Asians Living in American (MASALA) [59].

Physical and/or sexual violence were assessed as predictors of carotid IMT and plaques in two studies of middle-aged women [55, 56]. Mexican women who said they had experienced physical violence in adulthood had a greater cIMT and higher odds of carotid plaque [56]. Experiencing sexual violence was not related to subclinical CVD, but among women who reported physical violence, a longer duration of violence exposure was associated with greater cIMT in this sample. Thurston and colleagues reported similar findings in the Study of Women’s Health Across the Nation (SWAN), a longitudinal cohort study of mid-life women in the United States. Experiencing any sexual or physical abuse in adulthood was related to greater odds of carotid plaques but not greater cIMT [55]. Additionally, in the Pittsburgh Healthy Heart Study, participants who reported more psychologically demanding daily tasks had a marginally significantly greater change in carotid IMT and plaques over a period of six years, although this effect was only seen in participants who were not exposed to antihypertensive therapy [54].

Two studies used flow-mediated dilation (FMD), a measure of endothelial dysfunction. In the MESA study, chronic stress was related to lower FMD, indicating worse function [60]. Similarly, in a population of non-smoking, mid-life women, experiencing three or more traumatic events in adulthood (such as a serious accidents, disasters, illness, or injury) was related to lower FMD [61].

In sum, most commonly in the nine studies that examined subclinical CVD, general stress, including stressful life events and chronic stress burden, was positively related to subclinical outcomes, especially cIMT [54, 56, 57, 59] and FMD [60, 61].

### Subclinical CVD and social role-related stress and stressors

We found six studies that examined associations of social role-related stress or stressors with subclinical CVD in mid-life women. All were cross-sectional analyses, with assessment of role stress or stressors measured at a single time point, but two used progression of subclinical CVD as outcomes. As with the studies of general stress measures and subclinical CVD, results were mixed, especially for job stress. All six studies were rated ‘good’ on the QAT; three showed a positive relation between social role-related stress and stressors and increased subclinical CVD risk, and three studies reported null findings.

Four studies looked at measures of job stress and subclinical CVD and found limited evidence for an association. Charles et al. found that job strain was unrelated to FMD in employed participants in the MESA cohort [62]. Another analysis of data from the MESA study found no significant relationships between any occupational characteristics (based on participants’ occupation at Exam 1), including control, demand and interpersonal stress and progression of cIMT or plaques over a mean follow-up of 9.4 years after adjusting for CVD risk factors and indicators of socioeconomic position [63], with the exception of physically demanding jobs associated with a 15% increased plaque score among women. In the Brazilian Longitudinal Study of Adult Health, higher job control was cross-sectionally associated with lower cIMT in female civil servants, but women in passive jobs (low demand and low control) had significant greater cIMT than women with low strain jobs [64]. Job strain was also associated with higher central arterial stiffness (as measured by the Augmentation Index) but not carotid plaques or IMT in the Netherlands Study of Depression and Anxiety [58].

Regarding role-related stress other than job stress, two studies used ratings of social role quality to predict subclinical CVD. In SWAN the average level of role-related stress from up to four social roles (caregiver, employee, mother and relationship) was not associated with progression of CAC over two years. However, Black (but not white) women who rated their social roles as more rewarding had a reduced risk of having CAC progression greater than 10 Agatston units over two years [65]. In a cohort of 281 middle aged adults, participants with more negative interactions with spouses had greater cIMT than those who had fewer negative interactions, but an overall measure of global marriage quality (the Dyadic Adjustment Scale), measured at a single time point, was not related to cIMT [66].

There was limited evidence that job stress/strain was associated with subclinical CVD [58, 64] in working women, but negative spousal interaction was significantly associated with greater cIMT [66].

### Subclinical CVD and discrimination

Two recent studies, which we rated ‘good’ according to the QAT, examined the relation between discrimination and cIMT. In SWAN, a cumulative measure of unfair treatment over time was calculated by averaging scores on the Everyday Discrimination Scale that was administered to participants up to six times over 10 years of follow-up during mid-life. Higher scores on this measure were associated with greater cIMT but among Caucasian women only – not among African American, Chinese, Japanese or Hispanic women [67]. In the MASALA study, discrimination, also measured by the Everyday Discrimination Scale, was not related to cIMT in South Asian women [59].

### Summary: Stress and subclinical CVD measures

We identified a sample of mostly cross-sectional studies (13/15) that reported an association between stress or stressors and existing CVD diagnoses or concurrent subclinical CVD measures representing multiple pathologic features, including arterial stiffness, endothelial dysfunction and subclinical atherosclerosis. Overall about 2/3 of studies showed a positive relation between stress and subclinical CVD measures. The results for studies of subclinical CVD and general stress (including traumatic events, chronic burden and daily hassles and demands) were mixed, with most reporting significant associations for at least one stress/subclinical CVD combination [54, 56–61], and others finding no relationship between general stress and subclinical CVD [53, 55]. General stress was most consistently associated with cIMT and FMD, indicating a potential mechanism through endothelial function and remodeling. Although role-related stress was studied less than general stress, a study of marital interaction quality demonstrated a significant relationship between positive and negative interactions and cIMT. Four studies looked at job stress and found limited evidence that there was a relationship with subclinical CVD. Only two studies looked at discrimination and subclinical CVD, with perceived discrimination predictive of subclinical CVD in a prospective study of middle-aged women, but only among the white women in the study [67]. Only one study included a measure of arterial stiffness,and found that recent life stress was related to stiffer vessels, but not cIMT or plaques [58]. Arterial stiffness is believed to be a consequence of increased blood pressure exerting greater force on vessel walls. Additional longitudinal studies using measures of arterial stiffness as outcomes, such as pulse wave velocity, may shed light on the impact of stress-induced chronically elevated blood pressure on vasculature. Future studies of chronic stressors that use diverse, well-validated measures of subclinical cardiovascular disease will significantly contribute to the understanding of the pathophysiologic effects of stress on vascular disease and atherosclerosis.

## Future directions and discussion

### Summary of findings

In this review, we summarized the English language epidemiologic literature published since 2012 examining the relationship between stress and CVD in women, with a focus on the mid-life period. About 2/3 of studies showed a positive relationship between general stress exposures and increased CVD risk, of these 18 were ‘good’ and 6 were ‘fair’, and 1 was ‘poor’ according to the QAT ratings. Among the studies with null findings, 9 were rated ‘good’ and all 3 were rated ‘fair’. We targeted stressors that are relevant to the life experiences of mid-life women, including job stress, caregiving, marital quality and discrimination. The articles we reviewed supported the hypothesis that life stress and stressful events that occur in mid-life can impact women’s later-life risk for CVD events. Furthermore, there is evidence that this relationship can be observed during mid-life through measures of subclinical CVD, such as FMD, cIMT and CAC. In particular, physical and sexual violence, marital quality, and discrimination, three measures that are not common exposures in the overall stress-CV literature, showed potential for an impact on CV health for women. Job stress, on the other hand, is a commonly studied stress exposure, and findings related to this exposure were decidedly mixed. Caregiving stress has been discussed as an increasingly important exposure as the population ages, but we found few studies of this exposure in mid-life women have been published since 2012. Few studies identified significant sex differences in the effect of stress on CVD. The ability to find significant differences between men and women is related to the size of the sample population, which may be a limitation in the studies of CVD events which had small numbers of events. Our findings are consistent with prior reviews of the literature that supported the stress-CVD relationship in women [9, 10], and found that this relationship can be observed in populations that include mid-life women, and that subclinical measures of CVD have been observed to be related to stress in mid-life.

### Mechanisms of stress and CVD in mid-life

Studies of stress in mid-life women have the potential to provide a greater understanding of the mechanisms underlying the relationship between stress and CVD due to the physical and social changes associated with this transition. The hypothesized physiologic pathways by which chronic stress may lead to excess CVD risk include activation of the autonomic nervous system and hypothalamic-pituitary-axis, which leads to elevated inflammation and pre-clinical metabolic dysfunction [68]. These chronic low-level states of inflammation and metabolic dysfunction are proposed to contribute to the development and progression of atherosclerotic plaques and eventual CVD events. This process begins early in the life course, and progress occurs over decades. Cross-sectional studies, or studies that assess stress at a single time point, are limited in their ability to examine the relationship between stress and CVD risk at different stages of the life course. Longitudinal studies with measures of stress, inflammatory biomarkers, metabolic markers and subclinical CVD measures at multiple time points may provide insight into the underlying physiologic pathways through which stress contributes to elevated risk of CVD. The mid-life is an ideal time to study the mechanisms by which stress impacts CVD; although there are fewer events in women in this age group, it is when metabolic abnormalities and blood pressure dysregulation may start to develop. Noninvasive measures of atherosclerosis and arterial stiffness, two major pathways leading to CVD events, can also be done in this age group to estimate subclinical CVD risk.

Studies of stress and CVD risk conditions, such as the metabolic syndrome, diabetes and high blood pressure, are potential ways to study the stress-CV relationship in mid-life women. These conditions often develop during the mid-life, prior to clinical CVD events and are potential outcomes for epidemiologic studies in mid-life women, who rarely experience clinical events. Longitudinal studies with objective diagnoses and measures of these conditions can help shed light on potential mechanisms by which mid-life stress influences later-life CVD.

Finally, stress can influence participation in healthy or unhealthy behaviors such as smoking and physical activity in mid-life, which can lead to later-life CVD. There are many ways of measuring and quantifying health behaviors in epidemiologic studies, which makes it difficult to compare results across studies. Researchers interested in the relationship between stress and behaviors should consider using established guidelines such as the American Heart Association Life’s Simple 7 [69] as outcomes to facilitate comparison and utilization of results.

Stress is likely related to CVD through multiple pathways, including through CV risk conditions and unhealthy behaviors like smoking, both of which can occur in mid-life. Many of the studies of stress and CVD included in this review accounted for some of these mediators through adjustment as covariates, but not all studies could examine all mediators. Future research should consider whether covariates are confounders or mediators, and carefully interpret their results in this context.

### Other psychosocial factors

The primary aim of this review was to examine the effects of stress/stressors experienced by women in mid-life on their cardiovascular health. Other important stress exposures, including in different developmental periods, likely impact CVD risk in women but it was beyond the scope of this review to evaluate the impact of these other stressors. For example, it is recognized that stress experienced during childhood may influence later life CVD risk; moreover, stress experienced in adulthood may be a mediator of the relationship between early stress exposures and later life health outcomes. Two of our reviewed studies are relevant to this latter issue. One tested whether family strain in mid-life mediated the relationship between childhood mistreatment and myocardial infarction but found no support for this hypothesis [49]. Another reviewed study reported that trauma in adulthood was related to endothelial function, after controlling for childhood trauma [61]. In addition, we recognize that stress (including physiologic or behavioral reactions to stress/stressors) can mediate or moderate well-recognized relationships between other psychosocial factors such as negative emotions (depression, anxiety) or socioeconomic position and CVD risk. Indeed, stress and negative emotions may be a critical pathway by which socioeconomic status (SES) impacts health, although the evidence supporting this hypothesis is inconsistent [70]. A systematic evaluation of that literature is outside the purview of our review, but several studies that met our study criteria looked at the effect of socioeconomic status on the role of stress and CVD. In the REGARDS study, greater stress was associated with higher risk of acute CHD in low-income persons only [31]. Stress was hypothesized to be a mediator of the SES-CVD risk relationship in two studies, but there was little evidence of mediation in those studies [53, 64]. Finally, there is ample evidence that positive psychosocial factors, such as optimism, life engagement and psychological well-being, may be protective against the development of CVD [71, 72].

### Limitations

We attempted to comprehensively review all of the literature on stress and cardiovascular health and mid-life women over the past six years. Although psychosocial factors as predictors of CVD in women have been reviewed up until 2009 [9, 10], the transitional period in women ages 40–65 has never been exclusively reviewed to our knowledge. Examining stress in mid-life can shed light on the unique mechanisms of this transitional time and its impact on cardiovascular health in women. We used MeSH terms and multiple expressions to capture measures of stress relevant to mid-life women that are commonly used in epidemiologic studies; however, it is possible that we may have missed a group of studies that did not use our specific terms in their papers, including papers that focused on other types of stress exposures.

We included any study that included women in mid-life, which we defined as ages 40 to 65, even if it also included women outside of that age range, or men. There are significantly fewer studies focusing exclusively on mid-life populations of women, although there are several major cohort studies of mid-life women, including SWAN, the Nurses’ Health Studies and the Women’s Health Study which have contributed significantly to the understanding of women’s health in mid-life. Further studies of populations isolated to the specific period of mid-life are needed to better understand the unique exposures and health outcomes that women in mid-life experience. One challenge for these studies is the fact that statistical power often is limited to evaluate hard clinical endpoints since such events are much more common at older ages. Studying subclinical CVD in these populations could help solve this issue, where both stressors and subclinical CVD are measured multiple times throughout follow-up.

We identified few studies of non-job social role-specific stressors in mid-life women, an area that was identified in prior reviews as some potentially important sources of stress for women. The Chronic Burden Scale [21], used in several studies included in this review, contains individual items that ask about ongoing stress in three social roles (job, caregiver and relationship), as well as ongoing financial and personal health stress, but these separate items were rarely examined as separate predictors of CVD risk in mid-life women. Future studies of stress and women in mid-life should consider including validated role-specific assessments of stress, such as the Caregiver Self-Assessment Questionnaire [73]. This can aid in the development of potential interventions to address specific stressors for women in mid-life, such as workplace-based stress reduction programs, caregiver burden reduction interventions or improved support and resources for mothers or women in stressful relationships.

## Conclusion

The mid-life is a time of transition for women, which may result in increases in perceived stress and greater stress exposures. Stress related to life events, social relationships, work and discrimination can all contribute to increases in CVD risk in mid-life women, which can be measured objectively using subclinical measures, rather than waiting potentially decades to observe CVD events. A total of 37 studies from the past six years met our review criteria, the majority of which were evaluated to be of good quality; these studies looked at the impact of various stress exposures in mid-life on cardiovascular health in mid-life women. Most studies reported a positive relationship between greater stress in mid-life and cardiovascular health events in later-life. The findings for subclinical CVD outcomes were somewhat mixed, although carotid intima-media thickness, the most commonly-studied subclinical disease indicator, was related to stress in the majority of the studies in which it was assessed. Less consistent findings with other subclinical outcomes could be due to differences in the protocols used to measure subclinical CVD in these populations, the cross-sectional nature of most of the studies, as well as heterogeneity in the measures of stress. Furthermore, we found few studies of exclusively mid-life or female populations, and, while most studies that included men and women did not identify significant sex*stress interactions, some may have lacked power to detect sex differences especially if CVD events were rare. This review highlights the need for well-designed studies that utilize validated tools measuring specific stressors important to women in their transitional middle years, to more fully characterize and understand how stressful experiences in mid-life affect cardiovascular risk in women.

## Abbreviations

AF: atrial fibrillation
CAC: coronary artery calcification
CHD: coronary heart disease
CVD: cardiovascular disease
FMD: flow-mediated dilation
IMT: intima -media thickness
MASALA: Mediators of Atherosclerosis in South Asians Living in America
MESA: Multi-Ethnic Study of Adults
MetS: Metabolic Syndrome
MI: myocardial infarction
REGARDS: Reasons for Geographic and Racial Differences in Stroke
SES: Socio-economic status
SWAN: Study of Women’s Health Across the Nation
WSI: Weekly Stress Inventory
